# Multiparametric investigation of non functionalized-AGuIX nanoparticles in 3D human airway epithelium models demonstrates preferential targeting of tumor cells

**DOI:** 10.1186/s12951-020-00683-6

**Published:** 2020-09-10

**Authors:** Lucie Sancey, Odile Sabido, Zhiguo He, Fabien Rossetti, Alain Guignandon, Valérie Bin, Jean-Luc Coll, Michèle Cottier, François Lux, Olivier Tillement, Samuel Constant, Christophe Mas, Delphine Boudard

**Affiliations:** 1 Institute for Advanced Biosciences, INSERM U1209, CNRS, UMR 5309, Université Grenoble Alpes, 38000 Grenoble, France; 2 INSERM U1059, Laboratoire SAINBIOSE, équipe DVH/PIB, Faculté de Médecine, Université Jean Monnet, Saint-Etienne, France; 3 Université de Lyon, Saint-Etienne, France; 4 BiiGC EA2521, Saint-Etienne, France; 5 Institut Lumière Matière, CNRS UMR5306, Université Lyon 1, 69100 Villeurbanne, France; 6 SAINBIOSE, Inserm U1059, LBTO Team, Saint-Etienne, France; 7 CHU Saint Etienne, Hôpital Nord, UF6725 Cytologie et Histologie Rénale, St-Etienne, France; 8 NH Theraguix, 38240 Meylan, France; 9 Institut Universitaire de France (IUF), Paris, France; 10 Epithelix SARL, Geneva, Switzerland; 11 OncoTheis SARL, Geneva, Switzerland

**Keywords:** AGuIX^®^ nanoparticles, 3D human healthy and tumor airway models, Nanoparticle’s toxicity, Nanoparticle’s uptake, Tumor targeting

## Abstract

Liquid deposit mimicking surface aerosolization in the airway is a promising strategy for targeting bronchopulmonary tumors with reduced doses of nanoparticle (NPs). In mimicking and studying such delivery approaches, the use of human in vitro 3D culture models can bridge the gap between 2D cell culture and small animal investigations. Here, we exposed airway epithelia to liquid-apical gadolinium-based AGuIX^®^ NPs in order to determine their safety profile. We used a multiparametric methodology to investigate the NP’s distribution over time in both healthy and tumor-bearing 3D models. AGuIX^®^ NPs were able to target tumor cells in the absence of specific surface functionalization, without evidence of toxicity. Finally, we validated the therapeutic potential of this hybrid theranostic AGuIX^®^ NPs upon radiation exposure in this model. In conclusion, 3D cell cultures can efficiently mimic the normal and tumor-bearing airway epitheliums, providing an ethical and accessible model for the investigation of nebulized NPs. 
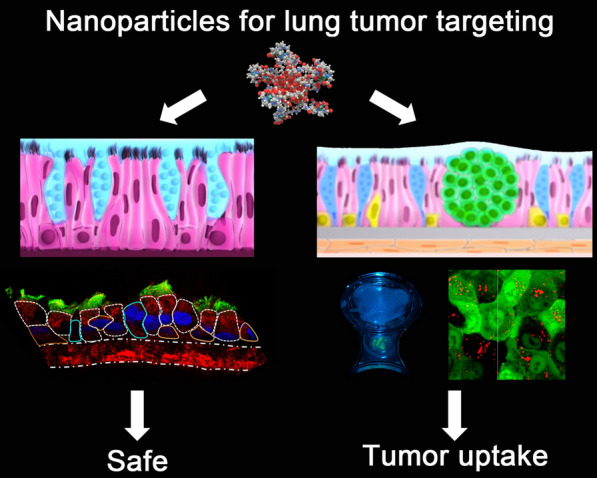

## Background

Non-small-cell lung cancer (NSCLC) is the principal type of lung cancer for the respiratory tract (85%), for which the standard treatment is surgery, especially for patients with early-stage NSCLC. However, some patients are not eligible for surgery because of medical comorbidities. In such cases, radiotherapy is an option that should be considered, in particular for early-stage, node-negative patients [1]. The standard of care recommendations promote the stereotactic radiation (SR) approach, a modality able to precisely deliver high-dose fractions to a small target or volume of disease, thereby reducing the dose or sparing healthy tissues [2]. Among SR approaches, image-guided radiation therapy (IGRT), intensity-modulated radiation therapy (IMRT), and volumetric modulated arc therapy (VMAT) constitute recent methods aimed at improving the efficacy of radiotherapy [3].

Despite these improvements, the main constraint is limiting toxicity in healthy tissues that should be spared. Therefore, the use of radiosensitizing agents containing high-Z elements such as gadolinium (Z = 64) [4–7], hafnium (Z = 72) [8], and gold (Z = 79) [9, 10], that specifically accumulate in the tumor has great potential for increasing the local effect of the dose deposited. From this perspective, gadolinium (Gd)-based nanoparticles (NPs) are of major interest, as they have both radiosensitizing characteristics and contrast properties for magnetic resonance imaging [11, 12]. The challenges of using radiotherapy for the treatment of lung diseases include gating the area of interest requiring radiation exposure. Such theranostic properties, *i.e.,* imaging ability and radiosensitization, suggest the strong potential of Gd-based NPs in the clinic [13].

To increase the lung tumor uptake of a drug, several administration routes can be used, including intravenous administration (IV) and inhalation [14, 15]. While the IV route allows vascularized tumor-specific and passive (through the EPR effect) targeting [16], it requires large volumes of drug, which increases the risk of toxicity/side effects and the treatment cost. In contrast, inhalation offers an interesting alternative that limits the administration volume, maximizes the local uptake and effect of the drug, and presents another pathway for targeting the tumor [17, 18]. In addition, inhaled drugs may cross the lung parenchyma and reach the blood stream. Because the drug is then circulating in blood, it may return to the tumor permeable blood vessels and accumulate by the EPR effect leading to two complementary tumor targeting mechanisms [12, 19].

In this context, we studied the toxicity and uptake, in terms of distribution and kinetics, of a Gd-based NP named AGuIX^®^, which has already been evaluated in phase 1b clinical trials (NCT02820454, NCT03308604) and is currently tested in phase 2 (NCT03818386 and NCT04094077) [20]. AGuIX^®^ NPs are ultrasmall NPs (≈5-nm) with MRI contrast and radiosensitizer properties and are considered theranostic nanodrugs for personalized medicine in oncology [21]. They have been tested in several preliminary in vitro and in vivo studies that evaluated their biotoxicity, biodistribution and biopersistence [4–6]. An in vivo study of localized AGuIX^®^ intrathecal instillation in mice showed increased tumor targeting and radiotherapy efficiency compared to systemic injection [12, 18, 19]. As well, AGuIX^®^ aerosolization may represent a promising theranostic approach for the treatment of lung tumors and metastases [22]. In order to mimic NP’s liquid aerosolization, we used two innovative 3D human models of the respiratory tract, one without tumor cells (MucilAir™/Epithelix) and one with A549 adenocarcinoma nodules (OncoCilAir™/Oncotheis) [23].

MucilAir™ is a fully differentiated and ready-to-use 3D model of human airway epithelium [24] made of primary human epithelial cells freshly isolated from nasal or bronchial human biopsies. MucilAir™ reproduces the morpho-functional characteristics of the original tight, polarized, pseudostratified, prismatic respiratory epithelia, with three types of cells: differentiated ciliated epithelial cells (> 50–60%), goblet cells (10–20%) that produce a part of the mucus secretion, and basal cells (remaining fraction) that enable epithelium renewal; the basal cells may be at an undifferentiated state or undergoing processes such as ciliogenesis [25–28]. The air–liquid interface of this in vitro model accurately reproduces the physiology of human airway epithelia, including the presence of a functional mucociliary system with ciliary beating and mucus secretion in a homeostatic state [24]. This biological interface is important to consider in pharmacological and nanotoxicological experiments [29, 30]. Similarly, OncoCilAir™ is a unique human in vitro 3D lung cancer model based on the coculture of human primary bronchial epithelial cells with incorporated A549-GFP tumor cells in the airway tissue, which facilitates observation and tumor growth. This model reproduces as closely as possible tumor invasion of a normal environment. In our case, A549 tumor cells were selected as the lung tumor model [31].

In the present study, we performed a bio-toxicological evaluation of Gd-based AGuIX^®^ NPs on airway MucilAir™ cultures, combining quantitative and qualitative NP uptake analysis through flow cytometry (FCM) approach, image acquisition (epi-fluorescence and confocal imaging), and ICP measurements [32–34]. NP exposure was achieved as restrained liquid exposure directly to the apical surface of the tissue with or without mucus interface, mimicking droplets-NP aerosolization and therefore the clinical approach. Second, we investigated and compared AGuIX^®^ NP distribution, and internalization by tumor and healthy cells in OncoCilAir™ cultures using multiparametric investigations. Finally, the radiosensitizing effect of AGuIX^®^ NPs was evaluated.

## Methods

### AGuIX^®^ NPs characteristics

The Gd-based nanoparticles are made of a polysiloxane network surrounding by cyclic chelates of gadolinium [35]. Their main characteristics are their ultra-small size with a hydrodynamic diameter of 3 ± 2 nm and a mass of approximately 10 kDa, a mean Gd/Si ratio of 10/40, and a strong complexation constant (log β_110_) for Gd (*i.e.* 24.78). The particles were developed for medical theranostic approaches, combining the contrast properties for MRI acquisition in T1 mode, and the radiosensitizing action of the high-Z element gadolinium (Z = 64). Different fluorescent dyes (FITC, Cy5.5, and Rhodamine-B (RhoB)) have been conjugated to the AGuIX^®^ for the biological investigations as previously reported [36], with a fluorescent conjugation yield of 1/600 to 1/250. A short characterization of the NPs can be found in Additional file 1: Figure S1. The AGuIX^®^ are freeze dried for long-term storage and can be solubilized in water and biocompatible solvent (NaCl 0.9%, as example) before administration.

### Culture of MucilAir/OncoCilAir™ tissues

MucilAir™ and OncoCilAir™ were produced by Epithelix and OncoTheis Companies respectively (see Additional file 1: Figure S2). A complete description of the cultures can be found on the websites (Epithelix, Geneva, Switzerland, http://www.epithelix.com and Oncotheis http://www.oncotheis.com). Upon receipt, bronchial MucilAir™ pool of donors cultures or OncoCilAir™ cultures (KRAS mutated) containing A549-GFP cells were transferred into 24-well plates filled with 700 µL of specific pre-warmed MucilAir™ or OncoCilAir™ culture medium. The tissues were routinely cultured at 37 °C and 5% CO_2_ in a saturated humidity environment (≥ 99%) in air–liquid interface (ALI) culture condition. Medium renewal was performed every two–three days. Trans-epithelial electrical resistance (TEER) was assessed as a standard indicator of tissues integrity. Measurements were conducted using an EVOM^®^ resistance meter and STX 2 electrodes (World Precision Instruments, Sarasota, USA) at least once a week, and before/after NPs exposure. The TEER values (Ω) were established by using the following formula: TEER (Ω·cm^2^) = (resistance value (Ω) − 100 (Ω)) × 0.33 (cm^2^), where 100 is the resistance of the insert membrane and 0.33 cm^2^ is the total surface of the 24-well plates culture insert. The TEER values described for well-preserved MucilAir™ cultures could be > 200 Ω·cm^2^ (well-preserved 200–600 Ω·cm^2^ [24] with optimal mean TEER 300–400 Ω·cm^2^ [25]), and for OncoCilAir™ around 100–200 Ω·cm^2^, the presence of tumor A549 areas decreasing the tightness of the epithelium, as reported by the company.

### AGuIX^®^ acute toxicity evaluation on MucilAir™

MucilAir™ AGuIX^®^ exposure has consisted of a liquid apical (air/liquid interface) or basal (liquid/liquid interface) 24-hour exposure, for final concentrations of 1 and 10 mM ([Gd^3+^]) NPs according to the experiments in their native or conjugated (FITC, RhoB, or Cy5.5) form; with kinetic monitoring up to 72-hour after apical and basal washing with PBS and addition of fresh medium in the basal compartment. AGuIX^®^ solutions have been prepared with specific MucilAir™ culture medium for basal exposure (Vf = 700 µL), or for apical exposure advisable vehicle saline solution of 10 mM Hepes, NaCl 0.9% and 1.25 mM CaCl_2_ (Vf = 30 µL). For apical exposure without mucus, the mucus was gently removed using a PBS washing step. All the bio-toxicity markers used for the analysis were assessed after the 24-h AGuIX^®^ exposure.

Morphological observations of cultures before and after AGuIX^®^ exposure were performed to evidence potentially figure modifications as decrease of ciliary frequency, detachment of cells, or apparition of cavity/hole into the insert using an inverted optical microscope (MOTIC AE2000 Trino).

Cell mortality was then evaluated by quantifying the lactate dehydrogenase (LDH) released from cells into the basal compartment with damaged membranes using the LDH Cytotoxicity kit plus (Roche Diagnostics, Mannheim, Germany), according to the manufacturer’s instructions. Optical density detection was performed using a microplate reader (Multiskan GO, Thermo Fisher Scientific Inc., Wyman Street, Waltham, USA) at 490 nm. The amount of the released LDH was reported as a percentage of cytotoxicity according to the following equation:

1 $${{\text{Cytotoxicity }}}\left( {\%} \right) = \frac{{\left( {{\text{OD\; of\; AGuIX\; exposed\; samples}} - {{\text{ OD\; of\; unexposed\; samples}}}} \right)}}{{\left( {{{\text{OD\; of\; positive\; controls }}} - {{\text{ OD\; of\; unexposed\; samples}}}} \right)}} \times 100$$

after subtraction for all values of blank control (OD of culture medium). The positive control corresponds to OD values after the complete lysis of control cells, with adequate buffer provided in the kit.

IL-8 pro-inflammatory response was assessed using a commercial enzyme-linked immunosorbent assay kit (Quantikine^®^ Human IL-8 Immunoassay, R&D systems Inc., McKinley Place NE, Minneapolis, USA). The optical density was determined according to the manufacturer’s instructions, using a microplate reader (Multiskan GO, Thermo Scientific) at 450 nm. A standard curve was established, and results were expressed as pg mL^−1^ of IL-8. A specific IL-8 positive control (cytomix stimulation: TNF-alpha at 500 ng/mL and LPS at 200 μg/mL incubated for 24-h in the basal culture medium) was used, according to the manufacturer recommendations [37]. It allowed the production of 4400 ± 510 pg/mL IL-8 (*p* = 0.028 compared to unexposed cells). Possible interaction of the NPs and the assays was checked to ensure the absence of false negative results.

### AGuIX^®^ permeability assessment on MucilAir™ barrier

To assess the apparent permeability (P_*app*_) of a compound across upper-airway epithelium of a respiratory tract, we specifically investigate the apical-to-basal transport of AGuIX-FITC in MucilAir™ tissues, using the same NP’s preparation as described in “AGuIX® acute toxicity evaluation on MucilAir^TM^” section. For this experiment, only inserts displaying a TEER around 300 Ω·cm^2^ were used. Atenolol (10 mM) and salicylic acid (100 μM) were respectively used as low and high chemical permeability molecules, and the values were compared with the one of AGuIX^®^ exposure [24, 38]. All compounds were diluted in HBSS transport buffer. Prior to incubation, all solutions were pre-warmed to 37 °C and the pH was adjusted to 7.4. Each condition was evaluated in triplicate. P_*app*_ was determined as follow. A pre-warmed HBSS donor solution (200 μL) was added to the apical compartment. A fraction of the donor solution was immediately withdrawn to determinate the effective initial concentration or fluorescence (AGuIX^®^-FITC). At the same time, 500 μL HBSS solution were filled into the basal acceptor compartment. Transport experiments were performed during 2 h at 37 °C. The fluorescent intensity of AGuIX^®^-FITC into apical and basal AGuIX^®^-FITC after this permeability experiment were measured with a fluorometer (Fluoskan Ascent, Thermo Fisher Scientific Inc., Wyman Street, Waltham, USA). The amount of atenolol and salicylic acid that permeated through the culture insert in both compartments was quantified by liquid chromatography-mass spectrometry (Aquity UPLC system) coupled with a Xevo TQ-D triple quadrupole mass spectrometer (Waters, Saint-Quentin-en-Yvelines, France). The permeability was finally calculated using the following equation:

2 $$Papp = \frac{V}{Ci \times A} \times \frac{Cf}{\Delta t}$$

where V is the volume of the donor compartment (cm^3^), C_i_ is the initial concentration of the compound (in g/L or M) or fluorescence intensity, A is the area of the insert (0.33 cm^2^), C_f_ is the final concentration of the compound in the acceptor compartment (in g/L or M) or fluorescence intensity, and ∆t is the duration of the experiment (in s). P*app* was expressed as cm·s^−1^.

### Functional monitoring of MucilAir™ cultures

Measurement of the Cilia Beating Frequency (CBF) was performed at room temperature (RT) by a dedicated set-up made of three parts: a camera (Sony XCD V60 Firewire), a PCI card, and a specific package of software. The CBF was calculated using CiliaX software (Epithelix, Geneva, Switzerland), and expressed as Hz.

The mucociliary clearance was monitored using a high-speed acquisition camera (Sony) connected to an Axiovert 200 M microscope (Zeiss, Jena, Germany). Microbeads (30 µm of diameter) were added onto the apical surface of the MucilAir™. Then, 30 s’ movies (3 movies/insert) showing the movement of the small beads will be taken and analyzed using the imaging software Image Pro Plus (Mediacy). The movement of the beads was tracked, and velocity of each particle was calculated to determine the speed of the mucociliary clearance.

### MucilAir™ phenotypic analysis by Flow Cytometry (FCM)

According to Epithelix company, MucilAir™ tissues were dissociated to surface insert with a trypsinization protocol to obtain a cellular suspension containing approximately 300 000 cells/insert. For each immuno-phenotyping, 100,000 cells were used. To avoid fixation/permeabilization steps inducing a significant loss of the intracellular AGuIX^®^ fluorescent signal, the cell type discrimination was performed with a vital CLCA1 staining (anti-CLCA1.PE-Cy5.5 human polyclonal goat antibody, #AC21-1575-16, Abcore, CA). CLCA1 is a membrane marker expressed in mediating calcium-activated chloride conductance for mucus production by goblet cells, whose specificity could be assessed in a previous MucilAir™ characterization study [25, 26]. This immunolabeling allowed a discriminant gating between the two differentiated cell types of CLCA1^+^ goblet population *vs*. CLCA1^−^ population, that included mostly ciliated cells and at last basal cells. Finally, for each sample, 50,000 cells were analyzed by flow cytometry (FCM), and the fluorescent signal was considered as quantitative. Intact cells were selected by a sequential gating using the FSC *vs.* SSC cytogram before selection according to specific CLCA1^+^ vs. CLCA1^−^ cell populations. Gain setting was established by running unstained and isotype controls cells (Lightning-link PE-Cy5.5 tandem conjugation kit to IgG isotype control antibody, Innova Biosciences). AGuIX^®^-FITC or GFP, green fluorescence, excited by 488 nm laser line was acquired through a BP 530/30 nm filter, PE-Cy5.5 excited by 488 nm laser line, through a 695/40 nm BP filter, and far red fluorescence of Cy5.5, excited by 633 nm laser line, through a 712/21 nm BP filter. Compensations were calculated by acquisition of fluorescent beads of each used fluorophore (Calibrat™ BD Biosciences, CA). Both percentage and geometric mean of fluorescence were collected. The results were expressed as the percentage of cells of interest, and geometric mean fluorescence intensity (MFI). FACS DiVa (BD Biosciences, CA) equipped with an argon ion and He–Ne lasers (Coherent, CA) was used. Data were analyzed with DiVa 5.03 software.

### Quantitative NPs’ uptake evaluation by FCM

Tumor cell population on OncocilAir™ model was ranked by FCM according to the GFP fluorescent signal intensity. The total cell population was therefore split into 3 sub-populations, *i.e*., normal healthy cells (GFP^−^), GFP^+^ and GFP^++^ tumor cells, expressing respectively low and high level of GFP protein. For each sub-population, the level of AGuIX^®^ uptake was determined and expressed as the  % of positive cells and MFI values. Finally, a relative uptake ratio values between tumor and normal areas was calculated as follow, after deduction of autofluorescence values:

3 $$AGuIX \; relative\; uptake\; ratio = \frac{{\left( {MFI_{GFP + , + + } } \right)}}{{\left( {MFI_{GFP - } } \right)}}$$

### Cell cycle analyzed by FCM

The cell cycle profile was determined according the double staining of DNA A-T specific vital dye Hoechst combined with propidium iodide (PI), a passive DNA intercalating dye. Hoechst fluorescence was collected after a UV excitation, while emission was collected through a 424/44 nm BP filter, and PI was excited at 488 nm, and its emission collected through a 695/40 nm BP filter. Dead cells and doublets of cells were excluded based upon PI positive staining, and specific gating drawing based upon Hoechst width signals *versus* Hoechst area signals respectively. Quiescent (G_0_/G_1_) and proliferative (S and G_2_ + M) cells were determined on the basis of linear quantification of Hoechst fluorescence.

### Microscopic observations of flat mounted MucilAir™/OncoCilAir™ tissues

#### Tissue preparations and observations

For observations of living tissues, the whole living MucilAir™/OncoCilAir™ tissues was removed from its cell culture insert and placed on a microscopic slide. A Viscoelastic System (Viscot, DuoVisc, Alcon) containing hyaluronate de sodium, chondroitin sulfate was added on the tissue for minimizing the cell stress during coverslip mounting. The tissue was then gently flattened using a large glass coverslip retained by adhesive tape.

#### Epifluorescence microscopy

By using an epifluorescence inverted microscope IX81 (Olympus, Tokyo, Japan) equipped with the CellSens imaging software (Olympus, Munster, Germany), the cells were visualized by phase-contrast, and tumor cells were highlighted by the GFP-fluorescence.

#### Confocal microscopy

The internalization of AGuIX^®^-Cy5.5 was relatively evaluated for A549-GFP tumor cells and healthy surrounding epi-airway epithelium using a FLUOVIEW FV1200 laser scanning confocal microscope (Olympus, Tokyo, Japan) equipped with the FV10-ASW4.1 imaging software. A 635 nm laser was used to excite the Cy5.5 dye, while the signal was collected using a long-pass filter λ > 650 nm.

#### Tumor-nodule area monitoring on OncoCilAir™

The growth of A549 tumor nodules tagged by GFP was observed on whole surface of OncoCilAir™ without any sample preparation, using a fluorescence macroscope MVX10 (Olympus, Tokyo, Japan) equipped with the software CellSens imaging systems (Olympus, Hamburg, Germany) or the macroscope system Leica equipped with the software Andor SOLIS (Oxford Instruments).

### Measurement of tumor area surface on OncoCilAir™

From images acquired with Olympus fluorescence macroscope at the time culture reception (i.e. 20–25 days after A549 cell implantation) and during their follow-up, an image processing using ImageJ platform has been performed (open-source program of the National Institutes of Health, http://rsb.info.nih.gov/ij/). Eight [8] bits raw images were first treated to remove the background and tumor edges were detected (edge filtering). Raw and edges images were combined to increase automatic detection of tumor areas. Detection of tumor areas was performed by applying a Reny threshold. Binary areas were sent to ROI manager to extract individually settings: area, perimeter, mean fluorescent intensity of all detected tumors. The mean fluorescent intensity signal of 100 over 255 was chosen to discriminate GFP^+^ and GFP^++^ populations. Mean signals under 100 were identified as GFP^+^, and GFP^++^ was attributed for values > 100. Individual temporal tracking of tumors areas and intensities were exported to GraphPad Prism7 for figures and statistical analysis.

### Additional nanoparticle observations by two-photon confocal microscopy

AGuIX^®^-RhoB (10 mM) were added to the apical phase of the cells during the indicated time. Two-photon microscopy was performed as described in Sancey et al. [39], using a LSM 7 MP (Zeiss, Germany) equipped with a 20 × water-immersion objective (NA 1.0; Zeiss) and ZEN 2010 software for detection of the NPs. Laser excitation was done at 800 nm with a Ti:Sapphire laser (Chameleon vision II; Coherent, UK). Fluorescence emissions were detected simultaneously by three non-descanned photomultiplier tubes with a 492/SP25 nm filter (Semrock, US) for blue autofluorescence and Hoechst emission, a 542/50 nm filter (Semrock, US) for green autofluorescence emission, and a 617/73 nm filter (Semrock, US) for AGuIX^®^-RhoB fluorescence emission. Autofluorescence and second harmonic generation of biological structures could also be collected in the 3 channels due to the presence of collagen, lectin and elastin as example. Confocal microscopy was performed using an LSM 510 (Zeiss Germany) equipped with a 40 × oil-immersion objective (NA 1.2; Zeiss). Laser excitations/emissions were 760 nm biphotonic/400–450 nm for Hoechst, 488 nm/500–550 nm for FITC/GFP, 543 nm/550–600 nm for Rhodamine-B, 633 nm/650–705 nm for Cy-5, respectively.

### Immuno-labeling for confocal microcopy

To discriminate the cellular organization of tumor areas from healthy tissue in OncoCilAir™ model, the sample was fixed in 4% paraformaldehyde (PFA) for 30 min at RT, then permealized in 0.5% Triton X-100 for 15 min at RT. The cells were stained with either ethidium bromide (FT-25810A, Molecular Probes) or phalloidin (Actin-stain™ 555, PHDH1, Cytoskeleton) for 30 min at RT, for nuclear and F-actin staining respectively. Phalloidin stained the cell outline of the respiratory epithelium. Before microscopic observations, the tissues were placed on a glass slide, covered with an anti-fading mounting medium (H1000, VECTASHIELD^®^, Vector) and gently flattened using a large glass coverslip retained by adhesive tape.

The inserts were incubated with AGuIX^®^ solution at 10 mM for 60 min to 72 h, in air/CO_2_ 5%, 37 °C environment. The inserts were cryofixed using liquid nitrogen vapor, and sliced at 8 µm. A solution of 5% goat serum in PBS was used to block any unspecific sites. To discriminate ciliated and goblet cells, a beta IV tubulin antibody was used (#T6793, 1/500, Sigma-Aldrich, France) and anti-mouse IgG-Alexa488 (#A11029, 1/2000, Life Technology, France). Beta IV tubulin is a major constituent of microtubules in motile ciliary axonemes. The sections were incubated with 1 mM Hoechst solution before mounting with the coverslip.

### Quantification of Gd content by inductively coupled plasma—optical emission spectrometry (ICP-OES)

Determination of the Gd content in the samples (apical and basal fractions, collected first after 24 h-exposure and secondly at 72 h end kinetic) was performed by ICP-OES analyses (Varian 710-ES) with a detection limit of 0.5 µg/L. The apical fraction was collected by gentle washing using saline solution, while basal fraction was sampling during medium renewal. Before measuring Gd concentration, 3 mL of aqua regia (mixture of acids: nitric and hydrochloric) were added to each fraction before heating for 3 h at 80 °C (SCP Science DigiPREP MS). After complete mineralization, the samples were diluted with HNO_3_ (5%, w/w) to reach a 50 or 10 mL-volume (respectively), and finally filtered at 0.2 µm for the measurements (*i.e*. a dilution factor of 500). The results were expressed as the total mass of Gd in the sample or its percentage relative to the administrated dose.

### Cultures radiation exposure and γH2AX labeling

The cultures were incubated or not with the AGuIX^®^ NPs (10 mM apical) 24 h before the radiation exposure, in presence of mucus. The x-ray exposure was performed at 4 Gy at 250 kVp; this dose was selected based on previous experiments. Similar experiments were performed on A549 2D cultures and the radiation exposure was conducted at 4 and 6 Gy. γH2AX labeling was performed on fixed cells as described in Kotb et al. [4], using anti-phospho-histone H2AX (Merck Millipore). Colony assay was also performed as described in Kotb et al. [4].

### Statistics

The Mann–Whitney test was used for the statistical analysis using Prism software for bio-toxicity and uptake experiments. Temporal tracking of tumors areas was compared using an ordinary one-way ANOVA with a Tukey test corrected for multiple comparisons as post hoc tests using a Graph Pad Prism7 suite.

## Results

### Biotoxicity evaluation of AGuIX^®^ NPs on MucilAir™

The synthesis of the gadolinium-based NPs AGuIX^®^ has been described previously; the main characteristics are available in Additional file 1: Figure S1 [21]. The administration of AGuIX^®^ NPs through the airways has been reported in vivo in mice with the hypothesis of both direct uptake and indirect vascular uptake after NP passage into the epithelium and blood stream [12]. Before investigating this phenomenon in details, the toxicity of AGuIX^®^ NPs was evaluated based on different and complementary biological parameters in healthy airway MucilAir™ inserts, according to previous nanotoxicological studies [40, 41]. The results summarized in Table 1 revealed that AGuIX^®^ NPs did not cause any loss of integrity of MucilAir™ epithelium or acute biological toxicity (see Table 1), even at the high dose of 10 mM, after a 24-hour direct exposure. However, a significant decrease in TEER values was observed for AGuIX^®^ NP after apical exposure (*p *< 0.05), regardless of the AGuIX^®^ NP dose or presence/absence of mucus. Yet TEER values had a similar profile in the saline vehicle negative control (NC) condition without mucus, indicating a stressful condition. This effect was certainly due to the condition of apical incubation with the saline solution, which acted as a cellular stress factor observed also in the NC condition especially without mucus. However, the integrity of the epithelium was not affected, even at the higher (10 mM) AGuIX^®^ NPs concentration, as the TEER values remained constant and clearly superior to the 200 Ω·cm^2^ lower threshold value. More interestingly, the AGuIX^®^ NPs did not have any impact on the functionality of the mucociliary system, based on mucociliary clearance and cilia beating frequency (CBF) analyses. Finally, the permeability (P*app*) with or without AGuIX^®^ NPs was comparable and > 10^−7^ cm·s^−1^, resulting in a stable P*app* value which appears even weaker than both control permeability molecules (high permeability molecule salicylic acid, P*app* = 1.91 × 10^−5^ cm·s^−1^; low permeability molecule atenolol, P*app* = 2.64 × 10^−6^ cm·s^−1^). So, an extremely weak MucilAir™ permeation was observed in presence of AGuIX^®^ NPs ≈ fivefold lower as compared to the one of atenolol which is an hydrophilic compound characterized by a permeability among the lowest established, involving passive paracellular way for transport across MucilAir™ barrier [38]. Due to the ultrasmall size of AGuIX^®^ NPs and previous investigations [19] these results suggest a transcellular AGuIX^®^ passage through the epithelium. The overall results of this morpho-functional evaluation demonstrated the absence of toxicity and the safety of AGuIX^®^ NPs under the tested conditions. Moreover, the presence or absence of mucus under apical exposure conditions had no impact on the toxicity of ultrasmall AGuIX^®^ NPs.

**Table 1:** Parameters evaluated on healthy MucilAir™ after a 24-h exposure to 1 and 10 mM AGuIX^®^ NPs revealed the absence of acute toxicity

Parameters	Unexposed with apical mucus	Unexposed w/o apical mucus	NC with apical mucus	NC w/o apical mucus	AGuIX 1 mM			AGuIX 10 mM		
					Apical exp. with mucus	Apical exp. w/o mucus	Basal exp.	Apical exp. with mucus	Apical exp. w/o mucus	Basal exp.
Morphological profile modifications	–	No	No	No	No	No	No	No	No	No
TEER (11 Ω·cm^2^)	463 ± 135	401 ± 70	465 ± 92	263 ± 61	297 ± 48.4	254 + 35	412 + 46	266 ± 40^#*^	261 + 22	422 + 81
LDH release (vs. unexposed cells)			< 5%	< 5%	< 5%	< 5%	< 5%	< 5%	< 5%	< 5%
1L8 production (pg/mL)	2630 ± 598	3550 ± 910	4046 ± 479	3690 ± 865	3273 ± 587	3720 ± 604	2810 ± 1038	2912 ± 615	3354 ± 1376	2726 ± 1038
Mucociliary clearance (μm/s)	ND	ND	33.0 ± 1.5	34.4 ± 2.5	ND	ND	ND	35.8 ± 1.9	35.6 ± 0.6	38.7 ± 1.4
Cilia beating frequency (Hz)	ND	ND	7.45 ± 0.57	6.28 ± 0.94	ND	ND	ND	7.77 ± 0.57	7.15 ± 0.6	6.65 ± 0.32
Permeability Papp (× 10^−7^ cm/s)	4.12 ± 0.7	3.61 ± 0.7	ND	ND	ND	ND	ND	5.91 ± 1.08	5.92 + 0.31	ND

Unexposed controls; NC: negative control treated with vehicle saline solution; without: w/o; ND: not determined. The experiment was performed on at least 9 distinct inserts. (*p < 0.05 *versus* unexposed cells; #*p *< 0.05 vs. negative control cells)

### Monitoring AGuIX^®^ NP behavior in healthy airway epithelium by FCM

**Fig. 1:**
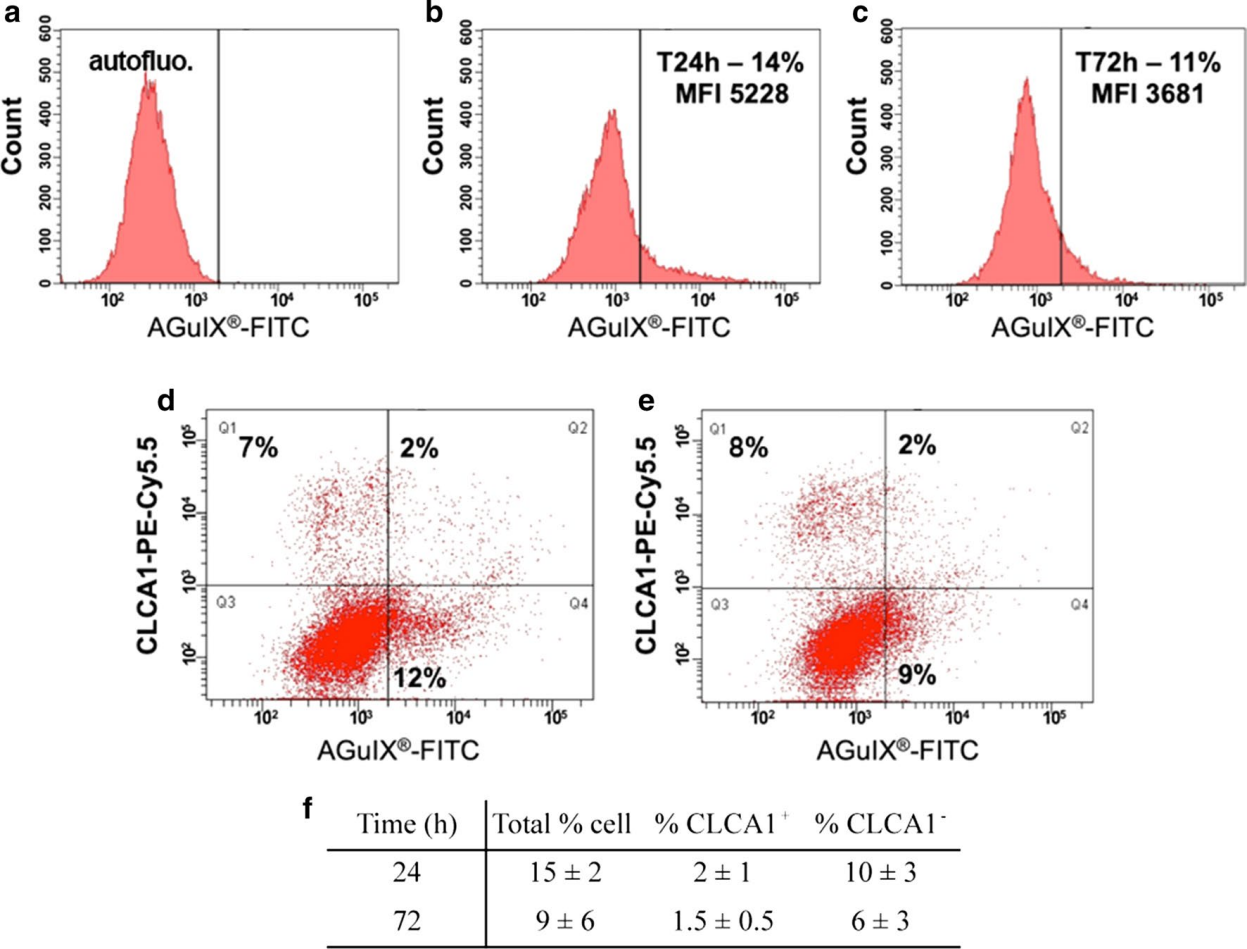
FCM quantification of AGuIX^®^ uptake in MucilAir™ cultures indicated a weak uptake in ciliated and basal cells. Representative histograms from one of the 3 experiments involving (**a**) control cells and AGuIX^®^-FITC-exposed cells for (**b**) 24 and (**c**) 72 h. The percentage of FITC-positive cells is presented as the geometric mean fluorescence intensity (MFI). Representative dot-plots of CLCA1 *versus* AGuIX^®^ signal at (**d**) 24 and (**e**) 72 h, with respectively 9% CLCA1-positive cells including 2% AGuIX^®^-positive *versus* 12% CLCA1-negative/AGuIX-positive cells and 10% CLCA1-positive cells including 2% AGuIX^®^-positive *versus* 9% CLCA1-negative/AGuIX-positive cells, and (**f**) the corresponding number of AGuIX^®^-positive cells expressed as % ± SD (mean of 3 experiments)

To mimic the direct administration of NPs by aerosolization, AGuIX^®^ NP exposure was restricted to liquid apical distribution in the presence of mucus (physiological condition) at the higher concentration of 10 mM in the next experiments (Fig. 1). The distribution of FITC-conjugated AGuIX^®^ NP was evaluated in the total viable population by FCM after 24 and 72 h of exposure (n = 3 independent experiments). FCM analysis showed that approximately 15 ± 2% of the MucilAir™ cell suspension exhibited a FITC fluorescence signal after a 24-h exposure (Fig. 1a, b, d, f). FITC fluorescence decreased heterogeneously with time, reaching almost 9 ± 6% at 72 h (Fig. 1c, e, f).

To identify the cell populations involved in AGuIX^®^ NP uptake, live cells were specifically labeled to discriminate ciliated cells from goblet cells. Of note, fixation and permeabilization were excluded to avoid major NP loss during the washing steps (> 50–80%, data not shown). The mucus-producing goblet cells were identified using an anti-CLCA1 antibody [25]. According to this characterization, 11 ± 2% of the live cells were CLCA1^+^ and considered goblet cells (Fig. 1d-e).

As described in Fig. 1f, during an additional 48 h of culture after the NP exposure period, the FITC signal of the NPs decreased from 15 ± 2% at 24 h to 9 ± 6% at 72 h. This reduction in FITC signal was mainly observed in the CLCA1^−^ cell population, which showed a decrease from 10 ± 3% to 6 ± 3%, while almost 2% of CLCA1^+^ cells presented a constant level of fluorescence. As indicated by the FCM analysis, AGuIX^®^ NPs were weakly internalized by healthy cells in the MucilAir™ airway epithelium and preferentially accumulated in CLCA1^−^ cell contingent (main ciliated population and basal cells) compared to CLCA1^+^ goblet cells.

### Confirmation of AGuIX^®^ NP distribution in the MucilAir™ model by confocal microscopy

**Fig. 2:**
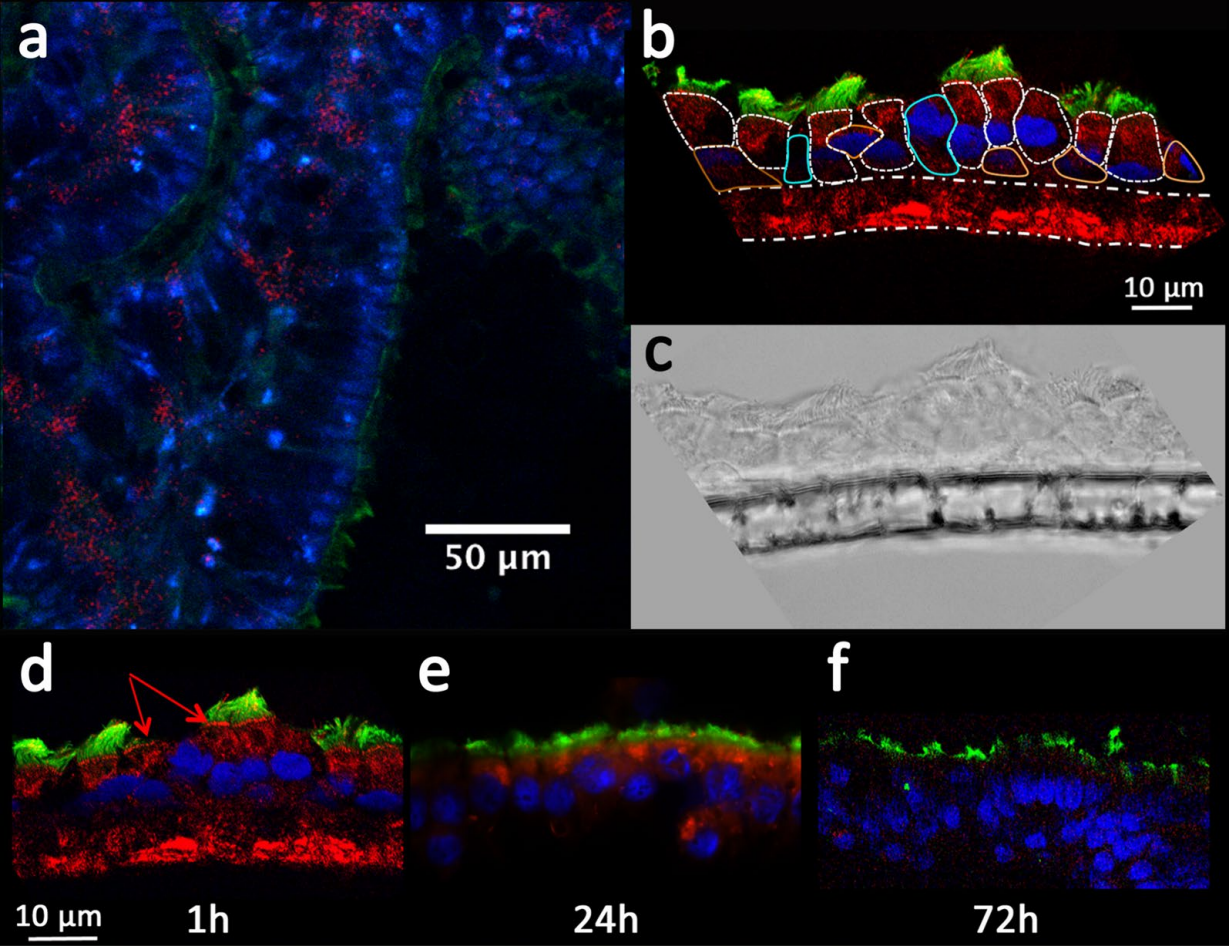
Overview of AGuIX^®^ NPs uptake and distribution on MucilAir™ by confocal microscopy evidenced the uptake in ciliated cells. **a** The AGuIX^®^-RhoB solution was added at 10 mM for 1 h and removed for observation using 2-photon confocal microscopy. Autofluorescence and second harmonic generation revealed structures such as cilia of ciliated cells (pseudocolored in green). Blue nuclear staining evidenced the nucleus. AGuIX^®^-RhoB NPs were heterogeneously distributed, as indicated by the red spots of cell vesicles. **b** Confocal layout showing the distribution of AGuIX^®^-RhoB NPs 1 h after apical exposure, with red arrows indicating AGuIX^®^ NP accumulation at the base of the cilia apparatus and **c** phase contrast of a MucilAir™ insert. The ciliated cells were identified by tubulin staining (green), the nuclei are pseudocolored in blue, and the NPs are represented in red. The ciliated cells are delineated by a dotted line (white), while the goblet cells are delineated with a blue continuous line, and the basal cells are delineated with an orange continuous line. The insert membrane is indicated by an irregular dotted line. **d**–**f** Confocal images of AGuIX^®^ NP distribution with time: 1 h, 24 h, and 72 h. The red signal of the NPs decreased during the 24–72 h kinetic period until it was almost completely eliminated at 72 h after exposure

Native MucilAir™ inserts were incubated with AGuIX^®^-RhoB and observed by intravital 2-photon microscopy (also see Additional file 1: Figure S3 for control condition). After a 1-hour incubation, the NPs were mostly observed in small cellular vesicles (Fig. 2a), away from the cilia, which was evidenced by the second harmonic generation of their components (collagen as an example). Labeling with a tubulin marker enabled the specific discrimination of the ciliary apparatus of the epithelium and the characterization of differentiated cilia cells. As illustrated in Fig. 2b, d, 1 h after apical exposure, the NPs accumulated in the apical pole of ciliated cells close to the cilia base and inside small cytoplasmic vesicles. Nonetheless, a strong RhoB signal was observed in the insert membrane itself, as indicated by the phase contrast layout (Fig. 2b-c). Therefore, at early incubation times (1 to 24 h), AGuIX^®^ NPs were mostly entrapped in the plastic membrane of the insert, between the apical and basal compartments, as well as in ciliated cells. After 24 h-exposure, goblet and basal cells were poorly positive (Fig. 2b), with preferential internalization into ciliated cells, confirming the cytometry data (CLCA1-negative cells). The microscopy and P*app* data were consistent with previous studies [19, 38] related to transient transcytosis mechanism; this mechanism allows AGuIX^®^ NPs to be carried from the apical compartment to the basal compartment, as evidenced by the progressive decrease in the RhoB signal (Fig. 2d–f). After 72 h, the fluorescence signal of AGuIX^®^-RhoB was almost undetectable by confocal microscopy.

### Quantification of AGuIX^®^ NP contents using ICP

Gd content was quantified by ICP measurements in the basal and apical fractions. As summarized in Table 2, the NPs were detected in both the apical and basal compartments after apical exposure, regardless of the presence of mucus. In particular, at 24 h, almost 60% of the initial AGuIX^®^ NP content remained in the apical fraction, while 14% was in the basal fraction, illustrating a reduced transcellular passage of NPs through the airway epithelium. In accordance with the microscopy observations on MucilAir™, very weak release of NP content was still observed at 72 h in the apical fraction of the same samples (< 0.5%), and the main content was measured in the basal fraction (≈ 6%). The remaining NPs were probably entrapped in the cells and in the plastic membrane of the inserts before passing into the basal compartment (see “Confirmation of AGuIX® NP distribution in the MucilAir^TM^ model by confocal microscopy” section). However, the limited number of cells (≈ 300,000) prevented us from achieving a reliable quantification of Gd from the cell pellets.

**Table 2:** Quantification of AGuIX^®^ NPs in the different fractions of MucilAir™ cultures after apical exposure to NPs at 10 mM demonstrated the fast clearance of healthy tissue

Conditions	24 h		72 h	
	Apical fraction (%)	Basal fraction (%)	Apical fraction (%)	Basal fraction (%)
With mucus	61 ± 20	14 ± 3	0.3 ± 0.1	7 ± 1
Without mucus	59 ± 19	13 ± 1	0.1 ± 0.1	5 ± 1

Gd was collected and measured by the ICP technique. The time point 72 h corresponds to 24 h exposure, followed by additional 48 h of culture. The results are presented as the percentage of the theoretical initial content

### Characterization of the OncoCilAir™ airway model

OncoCilAir™ cultures are similar to the MucilAir™ model but are enriched with GFP-expressing A549 human lung tumor cells cocultured with primary bronchial fibroblasts. The healthy OncoCilAir™ surrounding tissue is preserved, without alteration of the phenotype compared to MucilAir™ cultures, although the average TEER value was significantly lower than that in MucilAir™ cultures (156 ± 34 Ω·cm^2^ vs. 463 ± 135 Ω·cm^2^, n = 16/condition, *p *< 0.001) and was associated with a weaker ciliary beat frequency.

**Fig. 3:**
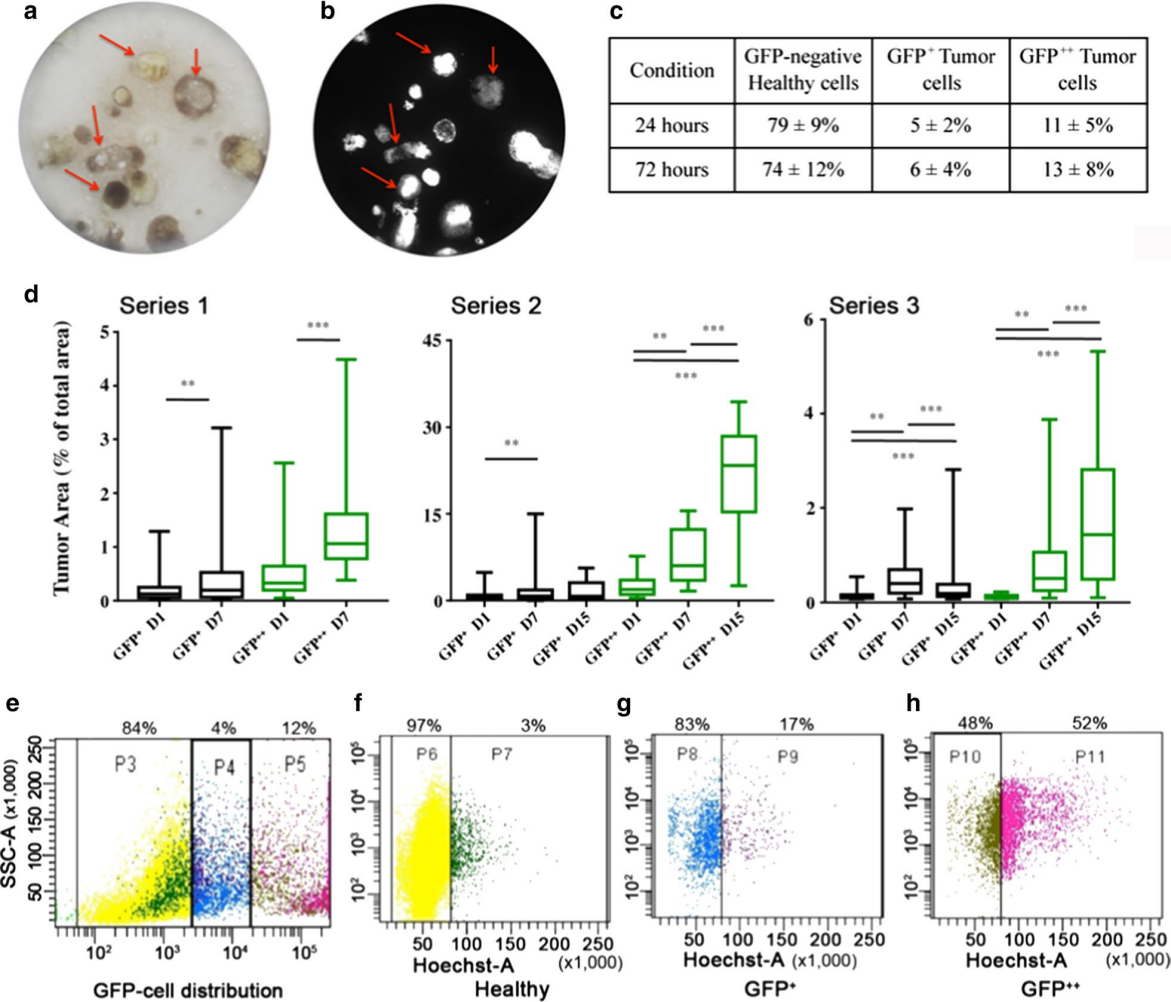
Characterization of the A549-OncoCilAir™ model revealed different cell proliferation profiles. Representative pictures under white light (**a**) and GFP-fluorescence (**b**) of the cultures, on a same insert. Tumor cell evolution according to GFP expression by FCM analysis during AGuIX^®^ NP’s exposure (**c**), monitored by microscopy for 2 weeks (d), and an example of FCM cell cycle analysis (**e**–**h**). **d** Boxplots (line: median, box: 25–75% percentiles, and whiskers: min–max) of the temporal evolution of relative tumor areas in 3 independent experiments. Bars indicate significant differences between groups (Tukey’s test, *p < 0.05, *p < 0.01, ***p < 0.001). **e** Example of the tumor cell subpopulations as a function of GFP status (n = 6). The 3 subpopulations containing no (P3, **f**), low (P4, **g**), or high (P5, **h**) levels of GFP fluorescence were separated and analyzed for cell cycle proliferation using Hoechst staining (n ≥ 9). The quiescent (P6, P8 and P10) and proliferative (P7, P9, and P11) subpopulations are shown. The different percentages of cells are indicated above each panel for this representative example

The cultures were observed by fluorescence microscopy over time to determine their characteristics and evolution. In these cultures, tumor cell aggregates organized as spheroids or more or less compact clusters, as illustrated by optical and macroscopic observations (Fig. 3a-b). The GFP signal allowed the discrimination of healthy cells (GFP-negative cells) and the identification of two different tumor subpopulations expressing low (GFP^+^) or high levels (GFP^++^) of GFP. The percentage of each GFP subpopulation varied with time as the tumors grew (Fig. 3c-h). A representative tumor evolution is described in Fig. 3d, which shows the tumor area recorded at day 0 (culture reception) and at one and two weeks later. The GFP^++^ cell population had a significantly faster proliferation rate than the GFP^+^ cell population, as indicated in Fig. 3d on 3 separate series, independently of the percentage of the tumor area in the culture.

FCM analysis performed for the 24–72 h AGuIX^®^ exposure indicated that GFP-negative cells accounted for 79 ± 9% of all cells at 24 h and 74 ± 12% at 72 h; the corresponding percentages were 5 ± 2% and 6 ± 4% for GFP^+^ cells and 11 ± 5% and 13 ± 8% for the GFP^++^ subpopulation (Fig. 3c–e, n = 6/condition, in triplicate).

To better describe the cell subpopulations with different GFP expression (Fig. 3e, n = 3), we characterized by FCM their proliferative capacity by studying the cell cycle (Fig. 3f–h). Cells in the proliferative phase made up a very weak percentage of the GFP-negative population (2.5 ± 0.5%) compared to the GFP^+^ (19 ± 2%) and GFP^++^ (47 ± 7%) populations, which were more proliferative. This cell cycle analysis demonstrates that first, the majority of the healthy cells are quiescent, which is expected for epithelial differentiated cells, and second, the GFP^+^ and GFP^++^ subpopulations are characterized by different proliferative capacities. Indeed, the GFP^++^ tumor cells were approximately 2.5 times more proliferative than the GFP^+^ cells, which may lead to differential AGuIX^®^ NP uptake according to proliferation rate.

### Distribution and quantification of AGuIX^®^ NPs in the OncoCilAir™ model

**Fig. 4:**
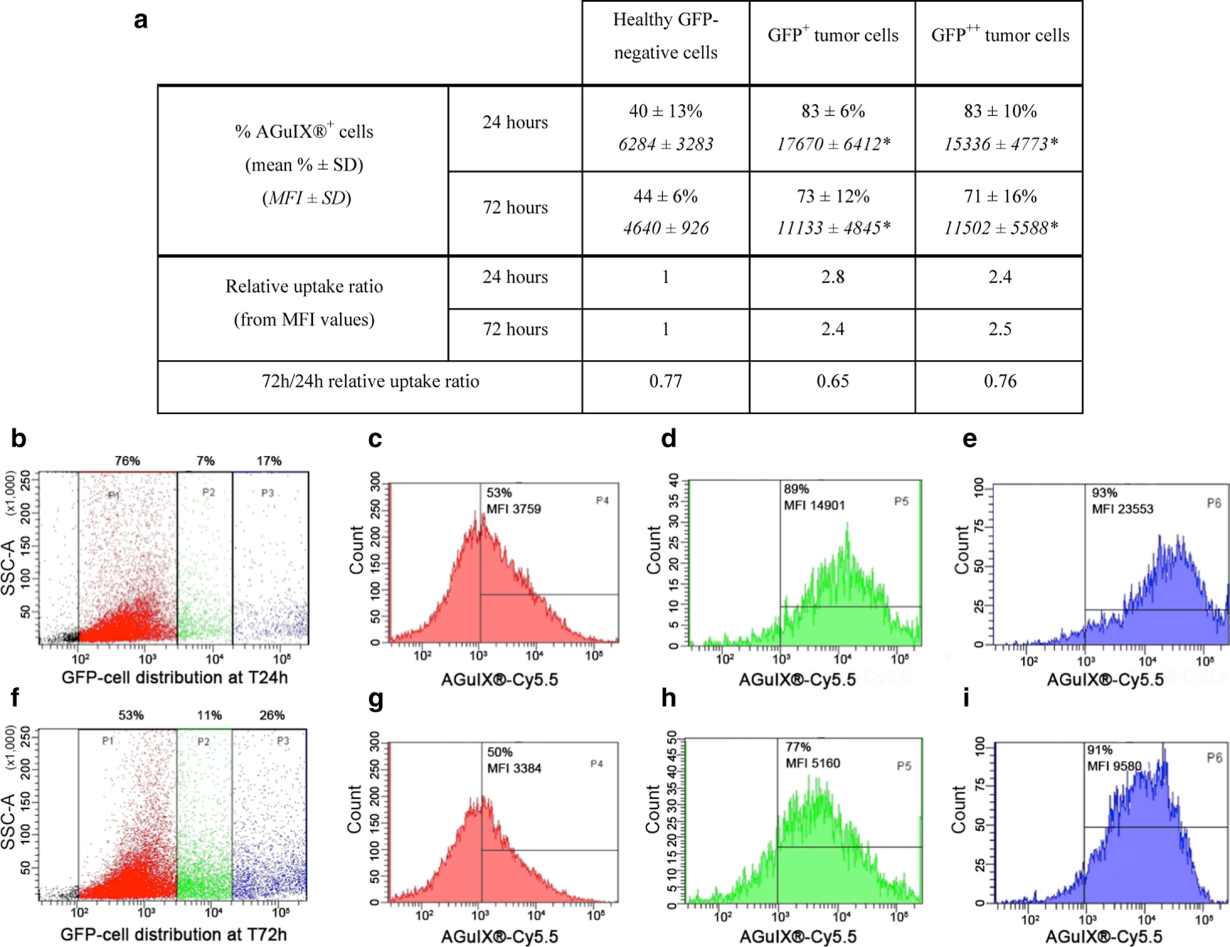
Illustration of AGuIX^®^ NP uptake measured by FCM as a function of time in the 3 OncoCilAir™ subpopulations. **a** Summary of AGuIX^®^ uptake with the percentage of AGuIX^®+^ cells and their corresponding MFI values presented after 24 and 72 h of NP exposure (n = 6/conditions, from 3 distinct batches of cell culture with and without mucus). The relative uptake ratios were calculated from Eq. 3. Statistical analysis was performed using the Mann–Whitney test (*p ≤ 0.05 compared to GFP-negative cells). The results **b**–**i** were obtained from samples of the 2nd series of cell cultures, presenting a fast tumor growth and an intense AGuIX^®^ uptake at T24h. FACS plots of AGuIX^®^ uptake (% of AGuIX^+^-cells and geometric mean MFI) according to the 3 gates (**b**, **f**) that discriminate GFP-negative (red channel; **c**, **g**), GFP^+^ (green channel; **d**, **h**) and GFP^++^ (blue channel; **e**, **i**) tumor cells at 24 h (**b**–**e**) and 72 h (**f**–**i**)

Similar to the experiments with MucilAir™, the distribution of AGuIX^®^ NPs was investigated in OncoCilAir™ (3 independent experiments), with or without apical mucus. FCM analysis allowed the observation of the 3 subpopulations over time, as shown in Fig. 3. As previously observed for the MucilAir™ cultures, the presence of mucus did not have an impact on AGuIX^®^ NP internalization on OncocilAir™ cultures (Fig. 4a, merged data from apical exposure with and without mucus). In addition, AGuIX^®^ NP’s exposure did not affect the production of IL-8 or LDH release compared to control (data not shown). After a 24-hour exposure (Fig. 4b–e), AGuIX^®^ NPs were preferentially detected in the tumor subpopulation, with ≈ 80% AGuIX^®^-positive/GFP-positive cells compared to ≈ 40% in normal healthy surrounding epithelial cells, and were associated with a significantly higher mean fluorescence intensity (MFI). The GFP tumor subpopulations possessed similar AGuIX^®^ uptake profiles, both in terms of percentage of positive cells and uptake (MFI values). The relative uptake ratio was 2.4 to 2.8-fold higher for tumor cells than for healthy cultures. The separate tumor proliferative capacity of GFP^+^ *versus* GFP^++^ cells did not clearly correlate with the uptake of NPs under our conditions.

After 72 h (Fig. 4f–i), the normal GFP-negative pool showed an equivalent percentage of AGuIX^®^-positive and about 20% decrease in AGuIX^®^-positive fluorescence intensity as compared to 24-h. Moreover, the NPs were released from a fraction of the tumor cells, as only ≈ 70% of the GFP^+/++^ cells were AGuIX^®^-positive, i.e., 10–15% less than at the 24-h time point. Such a reduction occurred concomitantly with a decrease in the MFI values about 30% both for GFP^+^ and GFP ^++^ populations confirming a partial release of NPs during this 48 h-time period (Fig. 4a). However, according to the AGuIX^®^ NP relative uptake ratios at 72 and 24 h for each population, the level of AGuIX^®^ internalization into GFP^+/++^ tumor areas remained 2.5 times higher than that in normal surrounding airway epithelium, as shown by the FCM quantification.

**Fig. 5:**
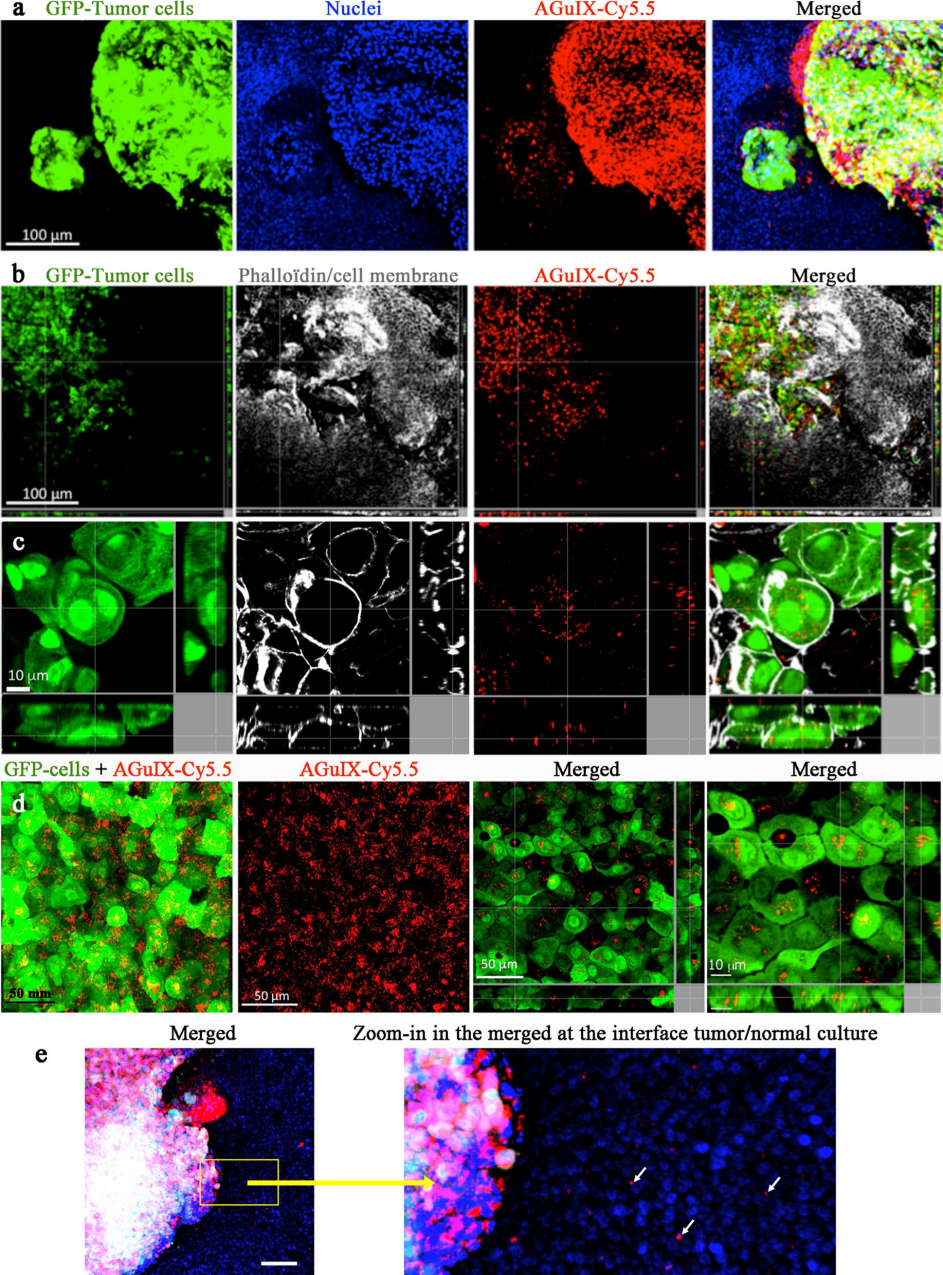
Representative confocal images of OncoCilAir™ inserts 24 h after AGuIX^®^-Cy5.5 exposure. **a** AGuIX^®^ NPs were preferentially observed in tumor areas. The GFP^+/++^ tumor cells (green) and healthy tissues were labeled with ethidium bromide to show nuclei (blue). AGuIX^®^-Cy5.5 NPs are represented in red. **b** Phalloidin staining (gray) revealed the organized healthy tissue compared to the chaotic tumor areas. **c** At higher magnification, the GFP/phalloidin colocalization confirmed AGuIX^®^ NP internalization inside tumor cells: the NPs were clustered into cytoplasmic vesicles. All images are orthogonal views presenting the XY, XZ and YZ planes. **d** Without cell fixation and permeabilization, AGuIX^®^-Cy5.5 NPs were also observed inside live tumor cells with clear evidence of NP internalization in cytoplasmic vesicles. The two first insets of **d** are 3D projection, while the two last are orthogonal views with different zooms. **e** A zoom-in at the interface between tumor and normal surrounding tissue evidenced the NP’s behavior. The arrows indicated the presence of NP clusters in the normal tissue. Scale bar: 50 µm

This efficient AGuIX^®^ NP uptake by tumor cells was confirmed by confocal microscopy. As illustrated in Fig. 5, AGuIX^®^ NPs were strongly internalized in tumor cells after 24 h of exposure, and the healthy surrounding environment remained almost unlabeled. The phalloidin labeling revealed the regular cell organization of the healthy airway environment, while the tumor areas were more chaotic and heterogeneous in terms of size and shape, as expected for tumor cell architecture. In Fig. 5a, one should note the NP accumulation in both the large compact and dense tumor area (right side) and the more diffuse tumor organization (left bottom). In addition, the higher magnification images showed NP internalization in small cytoplasmic vesicles (Fig. 5c, d, see also Additional file 1: Figure S4), as previously reported for tumor cell internalization in vivo, and in some peri-nuclear vesicles [4]. In a zoom-in at the interface between tumor and normal surrounding tissue, some internalized NPs were evidenced using increased contrast (Fig. 5e).

The ICP quantification of the NPs confirmed the strong passage of NPs through the OncoCilAir™ epithelium after apical exposure, as almost ≈ 20% of the particles were detected in the apical fraction at 24-h, while ≈ 40% were present in the basal compartment (Additional file 1: Table S1). This passage from the apical to the basal compartment was almost threefold higher in the OncoCilAir™ cultures with tumor cells than in the normal MucilAir™ cultures. At 72 h, the results were similar to those in the MucilAir™ cultures (Table 2), with ≈ 6/7% of the NPs released in the basal fraction and ≈ 1% in the apical fraction. The high NP passage into the basal compartment might be due to the presence of tumor cells, which affect the integrity of the airway epithelium. As indicated by the weak TEER values of the OncoCilAir™ epithelium (see “Characterization of the OncoCilAir^TM^ airway model” section), the culture’s integrity is modified by tumor areas, perhaps leading to efficient paracellular transport. This observation was correlated with cytometry analysis (Fig. 4a), where AGuIX^®^ uptake was quantified on OncoCilAir™ cultures for about 40% of GFP-negative healthy cells against 15% in MucilAir™ cultures (Fig. 1f).

### Radiosensitizing effect of AGuIX^®^ NPs on tumor cells and the OncoCilAir™ model

**Fig. 6:**
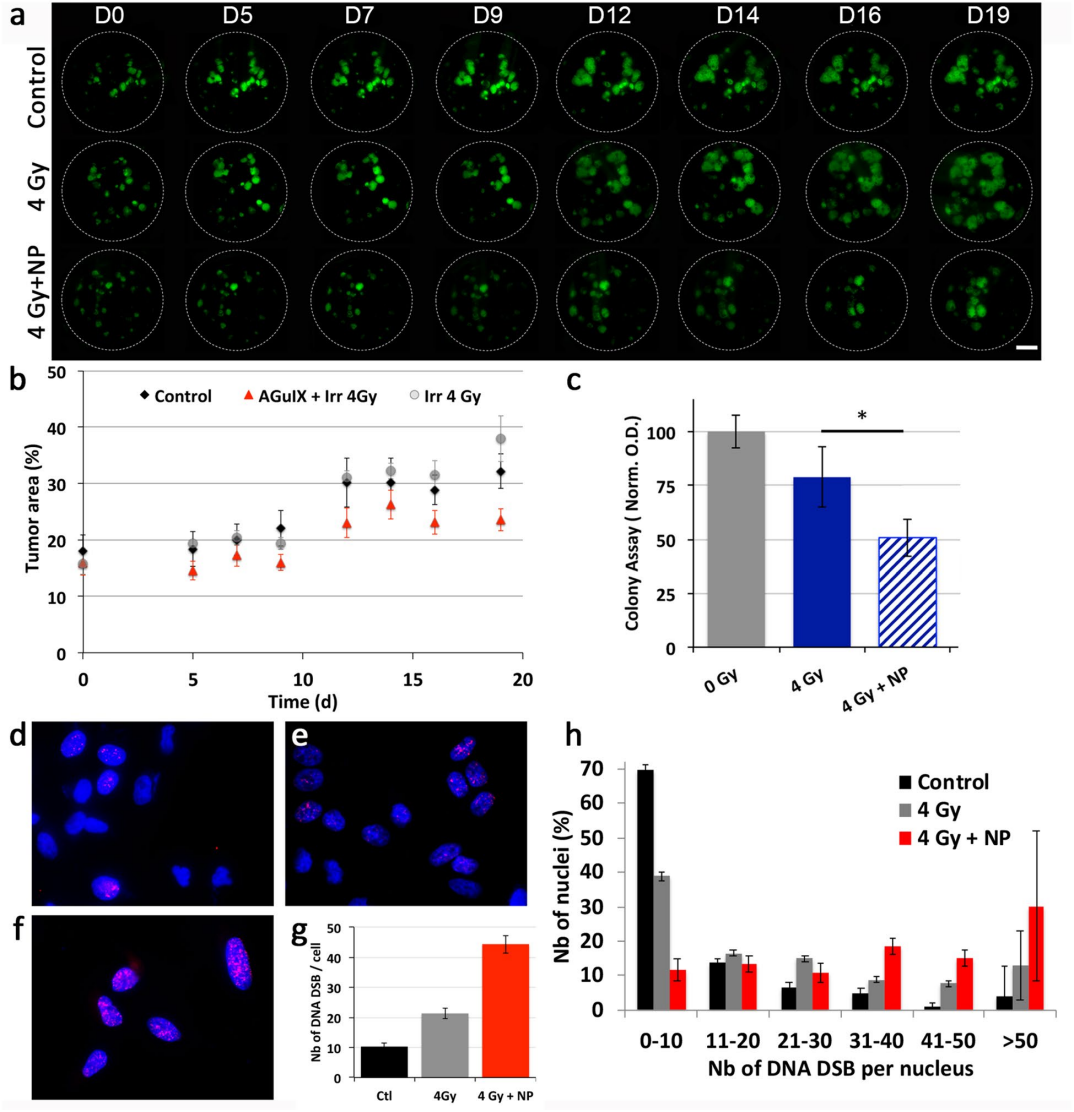
Radiosensitizing effect of the NPs was observed in OncoCilAir™ cultures. **a** The tumor growth development was monitored over time macroscopically (**a**) and the green tumor area was quantified **b** in the control group and the groups exposed to 4 Gy with and without NPs. Scale bar: 1 mm. **c** In vitro, A549-GFP cells were exposed to 4 Gy with and without NPs and seeded for 2D colony assays. The presence of NPs significantly reduced the number of colonies compared to irradiation alone. The cells plated in 2D were stained for γH2AX (in red) in the control group (**d**) and the 4 Gy groups without (**e**) and with NPs (**f**). DNA double-strand breaks (in red) (**g**), and nuclei (in blue) were counted (**h**) (n > 100/condition), revealing the increased amount of DNA damage in the combination treatment group

The radiosensitizing effect of AGuIX^®^ NPs was evaluated on OncoCilAir™ cultures after a 24-hour apical contact followed by exposure to 4 Gy radiation. Tumor growth (Fig. 6a) is expressed as the percentage of the area covered by tumor GFP-cells in the cultures. As indicated in Fig. 6b, the combination of AGuIX^®^ NPs and irradiation significantly reduced tumor growth compared to other conditions (*p *< 0.05 *vs*. control or irradiation alone). The overall tumor growth showed a mean reduction of 18% compared to the control. Subtherapeutic radiation with a single 4 Gy dose reduced tumor growth in the absence of NPs, although this reduction did not reach significance, before tumor growth relapsed (after day-10). In addition, LDH release and IL-8 production were monitored at days 1, 2 and 3 post exposure (data not shown). The results indicated the absence of cytotoxicity, with LDH release < 5% and counterpart IL-8 production compared to controls (4.000–4.500 pg/mL) under all conditions. One should note that the basal level of IL-8 production in unexposed OncoCilAir™ cultures was significantly increased compared to MucilAir™ cultures (*p* = 0.002*).

To better understand the biological impact of the AGuIX^®^ NPs, the experiment was repeated in vitro on GFP-A549 2D cultures alone after 4 Gy exposure. In 2D cell culture, the NPs in combination with irradiation significantly reduced cell viability compared to irradiation alone (Fig. 6c). Notably, the combination of NPs and 4 Gy exposure lead to similar cell growth as 6 Gy exposure alone in this 2D cell model (Additional file 1: Figure S5). In addition, DNA double-strand breaks were observed using γH2AX labeling (Fig. 6d–h). Exposure to 4 Gy radiation induced an increase in DNA double-strand breaks that doubled in the presence of NPs. In addition, the distribution of DNA double-strand breaks was determined under different conditions: such breaks were more numerous in the combined NP and irradiation treatment group than in the irradiation alone group.

## Discussion

From the perspective of bronchopulmonary cancer enhanced by radiation therapy, we investigated the safety, uptake, distribution, and radiosensitizing effect of ultrasmall gadolinium-based AGuIX^®^ NPs on human healthy 3D-cultured (MucilAir™) and human tumor-bearing epithelia (OncocilAir™). We used a multiple quantitative and qualitative approach to better compare and obtain information related to behavior.

As previously shown in the nasal MucilAir™ model [25], the bronchial pools of donors used to create MucilAir™ cultures mimic the morpho-functional characteristics of the human epi-airway with very good histological representation and mucus biological interface production. The intercellular cohesion of this epithelial model is principally ensured by the presence of tight junctions in the apical pole, which can be evaluated according to TEER and P*app* measurements [25, 38]. Here, the different toxicity parameters studied in MucilAir™ cultures, including TEER monitoring, LDH release, IL-8 production, permeability (P*app*), mucociliary clearance, and CBF did not show any acute toxicity, regardless of the presence of mucus, as previously observed for other preclinical models [6, 39, 42]. Interestingly, mucus did not affect the distribution of hybrid NPs. As previously reported, the penetration of small NPs inside respiratory mucus has been observed for particles up to 200/500 nm, while larger NPs (> 500 nm) mainly remain entrapped in the mucus [43–45]. NP penetration is related to NP size, and smaller NPs (< 60 nm size) were reported to pass through mucus pores. In addition, some particles, including those coated with PEG-moieties, might be muco-inert: these NPs do not interact with the mucus itself but are able to diffuse throughout mucus. In our conditions, the very small AGuIX^®^ NPs (< 5 nm) might possess such diffusing properties that facilitate their passage through mucus. Contrariwise, sub-micrometric CeO_2_ NPs aggregated in presence of mucus, reducing their transport across the respiratory mucosa [29].

The AGuIX^®^ NPs accumulated weakly in the healthy airway epithelium MucilAir™ and diffused into the basal compartment. NP diffusion might occur through transcellular way, as indicated by the microscopy observations, barrier integrity with high TEER values and according to AGuIX^®^ permeation analysis (P*app* measurements). Moreover, these observations also were in agreement with a toxico-kinetic MucilAir™ study, demonstrating that organic chemicals have a higher permeability when compared to inorganic compounds [38]. In addition, the very small NP size (< 5 nm) reinforces this observation. Furthermore, as demonstrated by the combined FCM and microscope analysis, ciliated cells are mainly involved in AGuIX^®^ NP uptake with a preferential occurrence at the apical pole of cilia cells, between motile cilia cells, with accumulation at the cilia anchoring area. This observation was in agreement with the principal function of surface exchange of this cell type. Such data are also in agreement with previous observations in vivo after the orotracheal administration of nanoparticles in mice bearing lung tumors. In this previous work, the nanoparticles strongly accumulated inside the tumors, while they were washed out of healthy tissues [19].

The distribution studies performed in OncoCilAir™ cultures confirmed a narrow NP uptake by healthy cells, uptake that was slightly higher as compared to the one of MucilAir™ cultures. Indeed, in OncoCilAir™ cultures, the A549 tumor microenvironment affects integrity of epithelial barrier, as indicated by the low TEER values, which might contribute to increase healthy cell uptake by promoting paracellular transport due to the reduction in intercellular tight junction bridges. Nevertheless, the NPs robustly accumulated in small cytoplasmic vesicles in tumor cells, as previously observed for subcutaneous tumors, for a prolonged time [4]: AGuIX^®^ NP fluorescence was still evident by FCM 72 h after NP exposure. The extended and high tumor cell uptake is an advantage for any clinical application in lung tumor enhanced radiotherapy: considering the unique administration of NPs in the airways, radiosensitizing NPs may stay inside the tumor region for several days to improve efficacy over several daily radiation exposures [6, 46].

The radiosensitizing effect of the gadolinium-based AGuIX^®^ NPs has been evidenced in various pathologies including lung tumors [4, 6, 46]. In this article, the radiosensitizing effect was observed for a single radiation exposure 24 h after the local administration of NPs, on OncoCilAir™ cultures. The combination of AGuIX^®^ NPs and 4 Gy irradiation has reduced significantly the surface tumor area compared to the irradiation alone. Interestingly, the 4 Gy radiosensitizing effect observed in the presence of NPs was similar to that measured after a 6 Gy exposure, on 2D-A549 cells (see Additional file 1: Figure S5). This result might indicate that a reduction in the exposure dose might be possible for a similar antitumor treatment for radiosensitive tumors or that radioresistant tumors might be more efficiently treated. In addition, the observed radiosensitizing effect might be increased in vivo, where the tumor lesions are vascularized. Indeed, in vivo, the AGuIX^®^ NPs might cross the epithelium and enter the vascular network, leading to further tumor accumulation though the EPR effect [4, 12]. The AGuIX^®^ NPs are not targeted NPs but passively accumulate inside tumor areas, as previously described in vitro and in vivo [4, 12, 13, 47, 48]. In this static in vitro culture system, the non-functionalized AGuIX^®^ NPs were preferentially internalized in tumor cells relatively to healthy cells. Besides EPR effect, the cellular mechanism responsible of the differential in vitro uptake should be further explored; we observed that AGuIX^®^ internalization was not related to tumor cell proliferation (Fig. 3), but might be due to active membrane transport and tumor cell permeability, as previously observed by macropinocytosis investigations [49]. This may indicate efficient AGuIX^®^ NP accumulation inside both highly and poorly proliferative tumors, similar to the cancer heterogeneity observed in the clinic.

To the best of our knowledge, this in vitro study reports for the first time the toxicity and distribution of NPs dedicated to theranostic applications, i.e., AGuIX^®^ NPs in both a healthy and an onco-airway 3D epithelium model. We demonstrate that healthy MucilAir™ cultures constitute a robust model for fundamental and applicable investigations of NP behavior due to homogeneity among different cultures, achieved by pooling cells from human donors [49]. Additionally, the impact of mucus, which constitutes an overriding biological interface for NP-cell interactions, might be studied in such an in vitro model [24, 25]. Under our conditions, the mucus had no impact on the uptake or the toxicity of the AGuIX^®^ NPs.

OncoCilAir™ cultures are a powerful in vitro model of 3D tissue for the assessment of anticancer therapy, as these cultures integrates A549 tumor cells representative of lung adenocarcinoma inside a healthy and functional airway tissue. While previous studies based on molecular compounds have already been performed on this type of model, this is the first study related to nanomedicine for oncology [30].

## Conclusion

For the first time, solid theranostic AGuIX^®^ NPs have been investigated in in vitro airway epithelium models with and without tumor areas. These valuable tools for bronchopulmonary investigations and anticancer therapies may bridge the gap between 2D cultures and human applications, allowing a reduction in animal testing while mimicking human behaviors. In these models, AGuIX^®^ NPs did not present evidence of toxicity, even at high local concentrations. The NPs preferentially accumulated in the tumor areas, where they were able to exert their radiosensitizing effect upon X-ray exposure. In the context of personalized medicine and enhanced radiation therapies for lung cancer, the administration of AGuIX^®^ NPs by aerosolization appears to be a promising and relevant approach.

## Supplementary information

**Additional file 1: Figure S1.** Main AGuIX characteristics. **Figure S2.** MucilAir™ and OncoCilAir™ histological and phenotypic characteristics. **Figure S3.** Complementary representative confocal images of OncoCilAir™ inserts 24 h after AGuIX^®^-Cy5.5 exposure. **Figure S4.** Radiosensitizing effect of the nanoparticles in combination with radiation exposure. **Table S1.** AGuIX^®^ NPs quantification by ICP-MS on OncoCilAir™ tissue.
