# Supplementary material for: Multiparametric investigation of non functionalized-AGuIX nanoparticles in 3D human airway epithelium models demonstrates preferential targeting of tumor cells
Source: J Nanobiotechnology. 2020 Sep 10;18:129. doi: 10.1186/s12951-020-00683-6 (PMC7488087; doi:10.1186/s12951-020-00683-6)
Supplement: Supplementary file 1 — Additional file 1: Figure S1. Main AGuIX characteristics. Figure S2. MucilAir™ and OncoCilAir™ histological and phenotypic characteristics. Figure S3. Complementary representative confocal images of OncoCilAir™ inserts 24 h after AGuIX®-Cy5.5 exposure. Figure S4. Radiosensitizing effect of the nanoparticles in combination with radiation exposure. Table S1. AGuIX® NPs quantification by ICP-MS on OncoCilAir™ tissue. [file 12951_2020_683_MOESM1_ESM.pdf]

Supplementary information

Multiparametric Investigation of non-functionalized-AGuIX Nanoparticles in 3D Human Airway Epithelium Models Demonstrates Preferential Targeting of Tumor Cells

Lucie Sancey[1], Odile Sabido[2,3], Zhiguo He[3,4], Fabien Rossetti[5], Alain Guignandon[6,3], Valérie Bin[2,3], Jean-Luc Coll[1], Michèle Cottier[2,3,7], François Lux[5,8,9], Olivier Tillement[5,8], Samuel Constant[10,11], Christophe Mas[11], Delphine Boudard[2,3,7].

[1] *Institute for Advanced Biosciences, INSERM U1209, CNRS UMR 5309, Université Grenoble Alpes, 38000 Grenoble, France*

[2] *INSERM U1059, laboratoire SAINBIOSE, équipe DVH/PIB, Faculté de Médecine, Université Jean Monnet, Saint-Etienne, France*

[3] *Université de Lyon, Saint-Etienne, France*

[4] *BiiGC EA2521, Saint-Etienne, France*

[5] *Institut Lumière Matière, CNRS UMR5306, Université Lyon 1, 69100 Villeurbanne, France*

[6] *SAINBIOSE, Inserm U1059, LBTO team, Saint-Etienne, France*

[7] *CHU Saint Etienne, Hôpital Nord, UF6725 Cytologie et Histologie Rénale, St-Etienne, France*

[8] *NH Theraguix, 38240 Meylan, France*

[9] *Institut Universitaire de France (IUF)*

[10] *Epithelix SARL, Geneva, Switzerland*

[11] *OncoTheis SARL, Geneva, Switzerland*

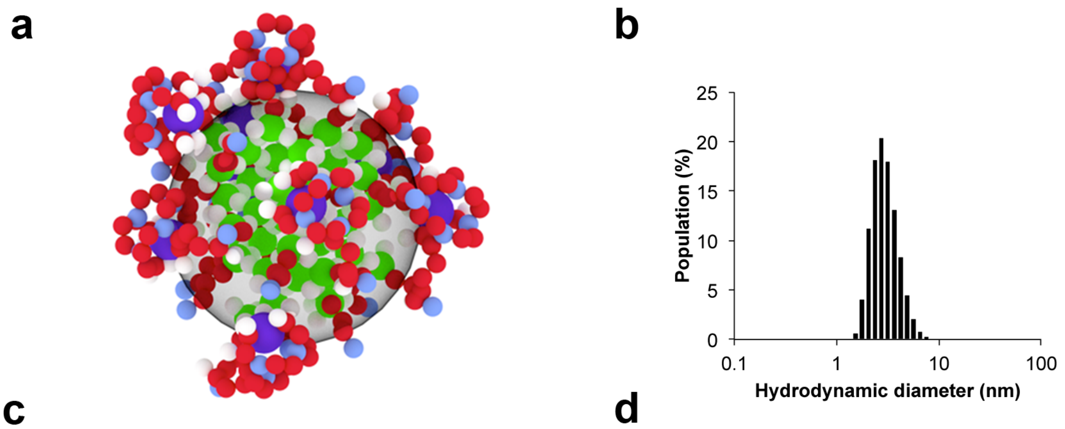

c

Main Characteristics of AGuIX Nanoparticles	
Chemical composition	$Gd_{10}Si_{40}C_{200}N_{50}O_{150}H_x$
Molar mass	10 ± 1 kDa
Size	3 ± 1.5 nm
Zeta potential	9.03 ± 5.5 mV

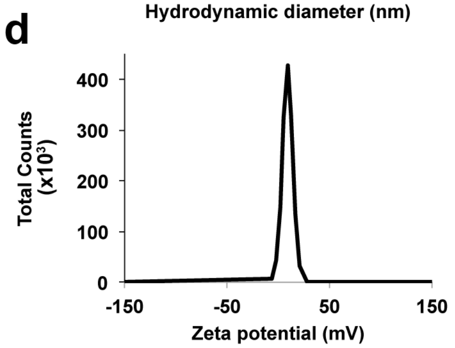

Figure S1: Main AGuIX® characteristics. (a) Representation of the nanoparticles with the following color code: green = Si, red = C, violet = Gd, blue = N, and white = H; (b) Hydrodynamic diameter of the nanoparticles; (c) Summary of the main characteristics; (d) Zeta potential of the nanoparticles. Adapted from Kotb et al. [6]

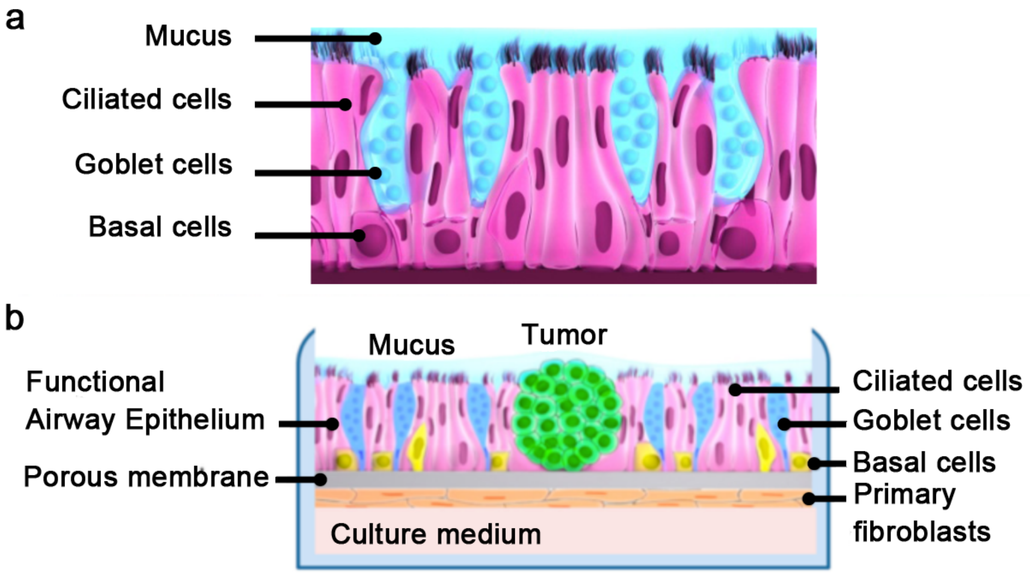

Figure S2: MucilAir™ and OncoCilAir™ histological and phenotypic characteristics. (a) MucilAir™ pool of donors cultures were used for this study, representative of the bronchial stage. Phenotypic characterization of goblet and ciliated cells was assessed with CLCA1 *versus* Beta4-tubulin markers respectively. (b) OncocilAir™ is a novel *in vitro* 3D human lung cancer model that combines a functional reconstituted human airway epithelium with A549-GFP^{+} lung adenocarcinoma cells and primary bronchial fibroblasts in a 24 wells transwell insert format.

Viable epithelial cells were observed by CMF using the anti-CLCA1.PE-Cy5.5 antibody (#AC21-1575-16, Abcore, CA) following supplier instructions: CLCA1^{+} (goblet population) *versus* CLCA1^{-} (ciliated and basal cells ongoing +/- ciliogenesis). For ciliated cells, anti-Beta4-tubulin (#T6793, Sigma-Aldrich, France) was used in addition for microscopy observations.

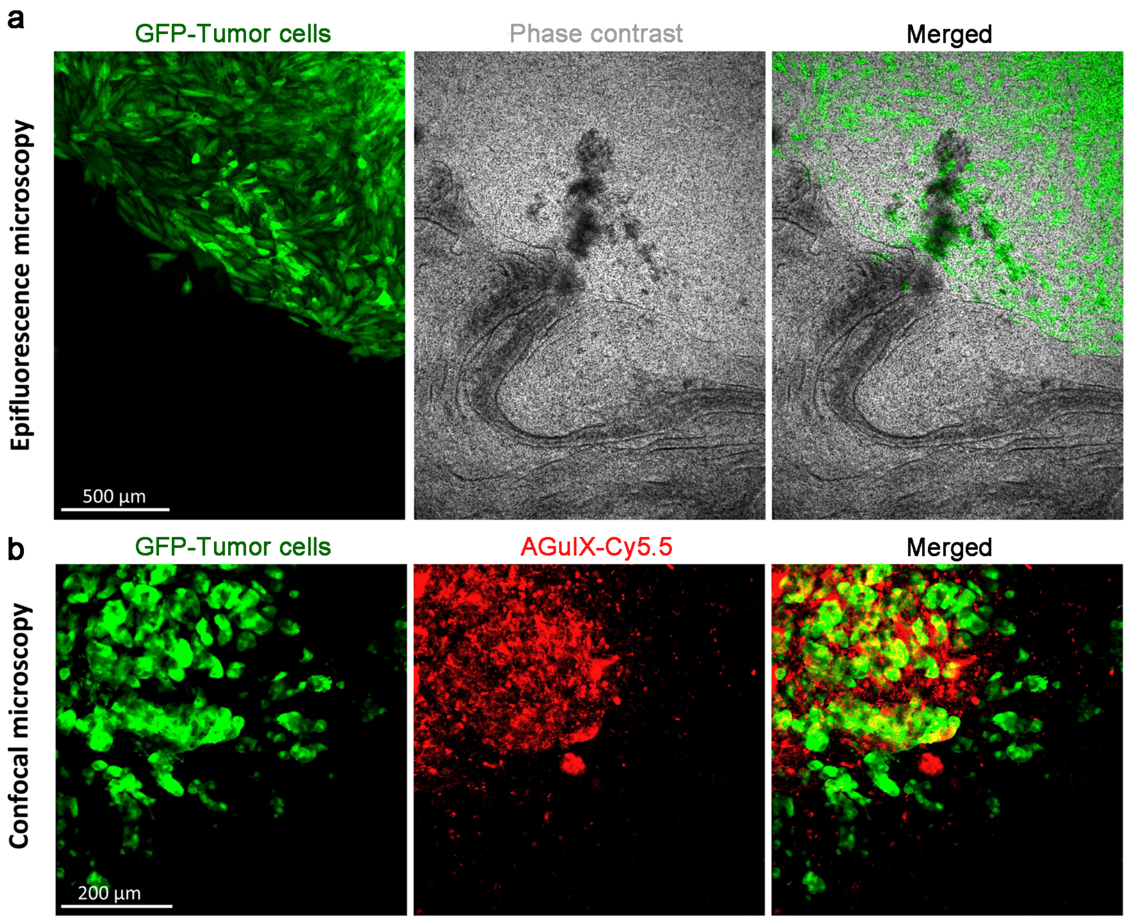

Figure S3: Strong and preferential AGuIX®-Cy5.5's internalization in tumor cells in viable OncoCilAir™ cultures. The OncoCilAir™ were mounted directly between a microscope slide and cover slip without any treatment to limit any artifacts. A Viscoelastic System (Viscot, DuoVisc, Alcon) was added onto the tissue for minimizing the cell stress caused the pressure between the slide and the cover slip. **(a)** Epifluorescence microscopy showing the integrity of the tissue in presence of both tumor GFP^+ and normal cells (Phase contrast). **(b)** The confocal microscopy pictures indicated a strong AGuIX®-Cy5.5's accumulation (red) in GFP tumor areas (green). All images represent the maximum intensity projection.

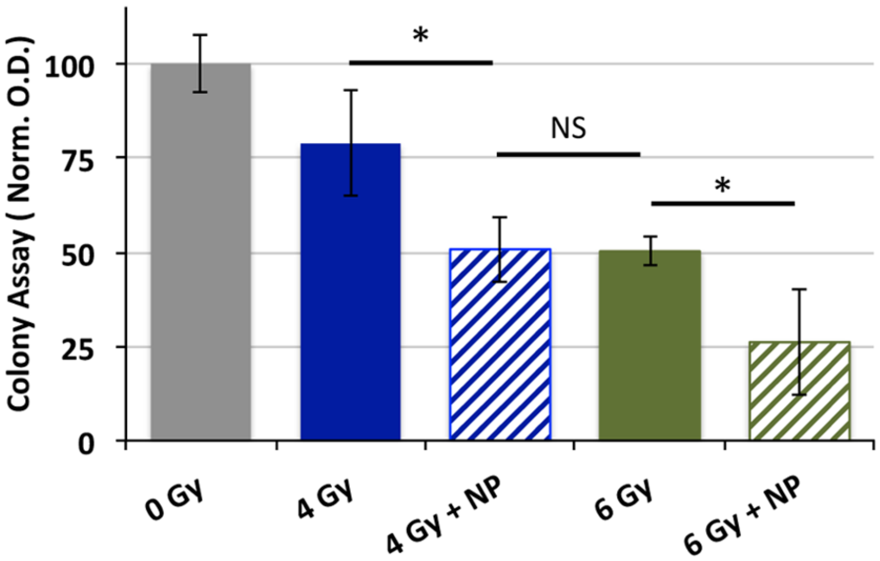

Figure S4: Radiosensitizing effect of the nanoparticles in combination with radiation exposure. The A549-GFP cells were exposed to 4 or 6 Gy, with and without NPs, and seed for 2D colony assay. The presence of NPs significantly reduced the number of colony as compared to irradiation alone.

Conditions	24-hours		72-hours	
	Apical fraction (%)	Basal fraction (%)	Apical fraction (%)	Basal fraction (%)
With Mucus	20 ± 8	37 ± 18	1 ± 0.3	4.5 ± 0.5
Without Mucus	19 ± 9	39 ± 17	0.5 ± 0.4	4 ± 0.4

Table S1: AGuIX® NPs quantification by ICP-MS on OncoCilAir™ tissue, after apical exposure at 10 mM with or without mucus, compared to the initial Gd content (n=3).
